# Renal hyperfiltration with and without metabolic syndrome: differential implications for cardiovascular events, kidney failure, and mortality

**DOI:** 10.3389/fnut.2025.1652372

**Published:** 2025-10-14

**Authors:** Yu Ho Lee, Dae Kyu Kim, Jin Sug Kim, Su Jin Jeong, Kyung Hwan Jeong, Hyeon Seok Hwang

**Affiliations:** ^1^Division of Nephrology, Department of Internal Medicine, Kyung Hee University Medical Center, Kyung Hee University, Seoul, Republic of Korea; ^2^Statistics Support Part, Medical Science Research Institute, Kyung Hee University, Seoul, Republic of Korea

**Keywords:** renal hyperfiltration, metabolic syndrome, cardiovascular event, end-stage kidney disease, all-cause mortality

## Abstract

**Background:**

Renal hyperfiltration (RHF) and metabolic syndrome (MetS) share common pathophysiology and are both associated with adverse clinical outcomes. However, their combined impact remains unclear.

**Methods:**

In total, 278,552 propensity score-matched individuals were enrolled in the Korean National Health Insurance Service database (2009–2011). Participants were divided into four groups based on RHF and MetS status, and cardiovascular (CV) events, end-stage kidney disease (ESKD) progression, and all-cause mortality were evaluated.

**Results:**

Compared to non-MetS with normal renal filtration (NRF), MetS with NRF was associated with a significant increase in the risk of CV events, which was further amplified when combined with RHF (adjusted HR = 1.44, 95% CI = 1.35–1.55, P for interaction = 0.047). Patients with RHF exhibited more pronounced increases in the HRs for CV events than those with NRF as the number of dysfunctional metabolic components increased (P for interaction = 0.019). The risk of ESKD progression was not increased in non-MetS with RHF; however, it was significantly higher in patients with MetS alone and highest in those with both MetS and RHF (adjusted HR = 3.23, 95% CI = 1.61–6.47). The risk of all-cause mortality was elevated in patients with RHF or MetS alone and highest in those with both RHF and MetS (adjusted HR = 1.41, 95% CI = 1.31–1.52).

**Conclusion:**

The clinical significance of RHF differs based on MetS status, with their coexistence posing the highest risk for CV events, ESKD progression, and all-cause mortality. A synergistic interaction between RHF and MetS was evident in the risk of CV events.

## 1 Introduction

Metabolic syndrome (MetS) is a common disorder characterized by a cluster of metabolic risk factors, and its prevalence continues to increase (1–3). MetS precedes major morbidities such as diabetes, hypertension, and dyslipidemia and is a key risk factor for cardiovascular (CV) complications and all-cause mortality (4, 5). MetS is associated with an increased risk of renal injury, with metabolic burden linked to a higher incidence of chronic kidney disease (6, 7). Therefore, metabolic dysfunction is central to the pathogenesis of CV and renal diseases, emphasizing the need for comprehensive risk assessment and intervention.

Glomerular filtration is the process by which kidneys filter blood, removing excess fluids and waste products to form urine. Renal hyperfiltration (RHF), defined as an increased glomerular filtration rate above normal levels, is an early manifestation of kidney disease and often occurs in diabetes and hypertension (8, 9). Key pathophysiological mechanisms underlying RHF include renin-angiotensin system activation, heightened sympathetic nervous system activity, and endothelial dysfunction, contributing to increased glomerular permeability and capillary hydraulic pressure (10). These pathophysiological processes are also common in individuals with MetS, which has been strongly associated with the development of RHF, even before MetS-associated overt cardiometabolic complications typically linked to MetS emerge (11, 12).

Considering the shared pathophysiological mechanisms and overlapping clinical associations of MetS and RHF, a deeper understanding of their combined impact on long-term outcomes is warranted. However, how RHF affects adverse clinical outcomes in relation to MetS status remains unclear. We aimed to investigate whether the risks of CV events, kidney failure, and all-cause mortality differ according to the presence or absence of MetS and RHF, using a large national health screening dataset.

## 2 Materials and methods

### 2.1 Study participants

This study used the National Health Insurance Database (NHID) in Korea, a comprehensive data repository covering the entire population provided by the National Health Insurance Service (NHIS). Detailed information on the NHID structure and variables is described elsewhere (13–15). The NHID contains clinical demographics, diagnosis codes (International Classification of Diseases, 10th edition [ICD-10]), insured medical services, and data from the National Health Screening Program, which includes health questionnaires and laboratory tests obtained annually for non-office workers and biannually for office workers (16). The institutional review board approved this study (no. 2022–02-045), and the use of the NHIS database (NHIS-2020-01-470) was approved. This study was conducted according to the Strengthening the Reporting of Observational Studies in Epidemiology (STROBE) statement, and the study protocol complied with the Declaration of Helsinki.

**Figure 1:**
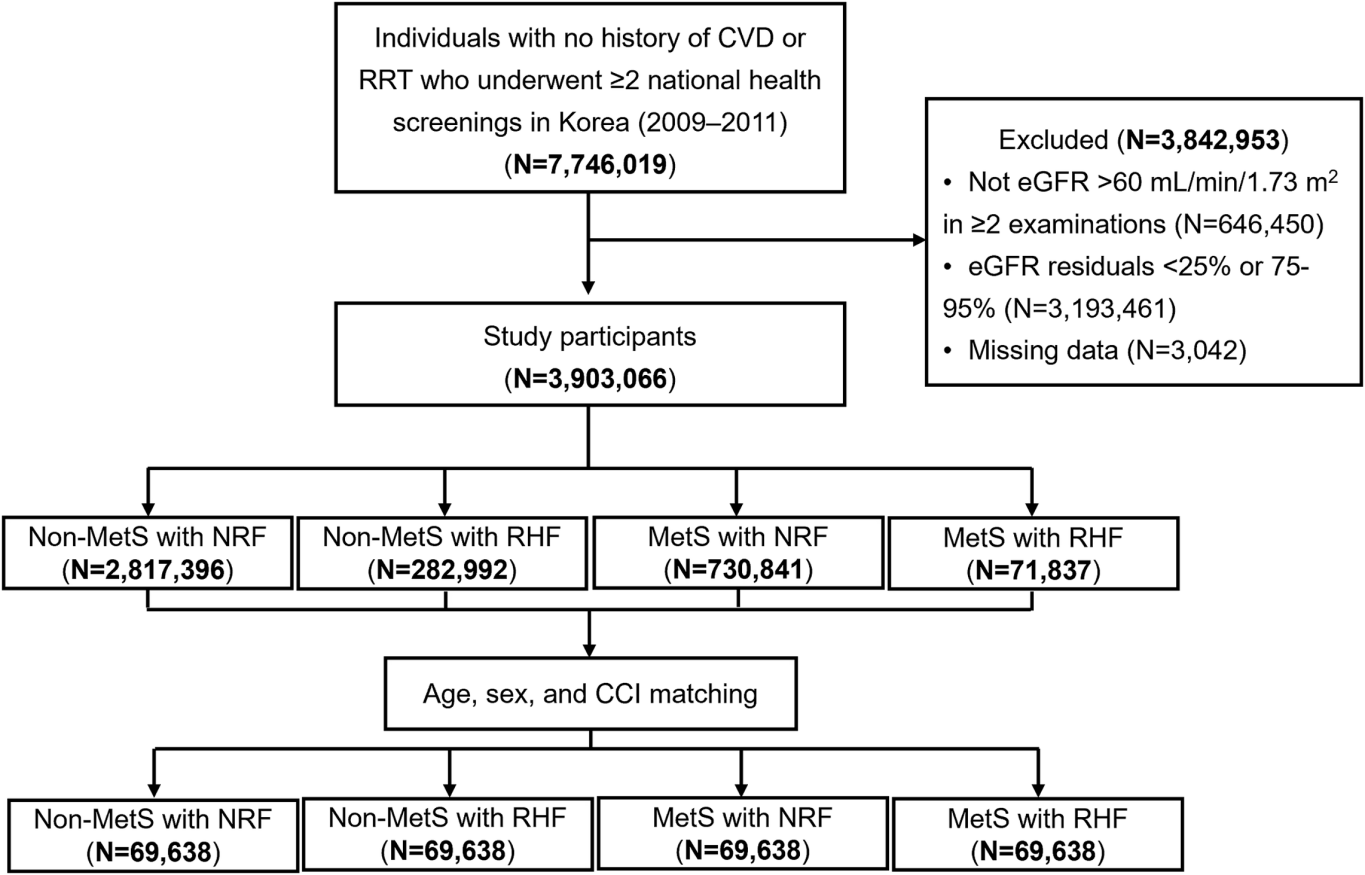
Flowchart of study participant selection. CVD, cardiovascular disease; RRT, renal replacement therapy; eGFR, estimated glomerular filtration rate; MetS, metabolic syndrome; NRF, normal renal filtration; RHF, renal hyperfiltration; CCI, Charlson comorbidity index.

A flowchart of the participant selection strategy is illustrated in Figure 1. This study screened 7,746,019 individuals aged >19 years who underwent ≥2 health screenings between 2009 and 2011 and had no history of CV disease (ICD-10 codes I20–25 or I60–69) or renal replacement therapy (identified as insurance codes for dialysis or transplant). Participants were excluded if they did not have estimated glomerular filtration rate (eGFR) > 60 mL/min/1.73 m2 in ≥2 health examinations (*n* = 646,450), residuals of eGFR below the 25th percentile or between the 75th and 95th percentiles (*n* = 3,193,461), or missing key variable measurements (n = 3,042). Therefore, 3,903,066 individuals were included in the study.

### 2.2 Assessment of metabolic syndrome and renal hyperfiltration

MetS was defined based on the modified National Cholesterol Education Program (NCEP)-Adult Treatment Panel (ATP) III criteria (17). Individuals were diagnosed with MetS if they had ≥3 of the following risk factors: abdominal circumstance >90 cm for men and >80 cm for women, high fasting glucose (≥100 mg/dL and/or use of antidiabetic drugs), elevated blood pressure (≥130/85 mmHg and/or use of antihypertensive drugs), hypertriglyceridemia (≥150 mg/dL or treatment for elevated triglycerides and/or use of lipid-lowering drugs), and low high-density lipoprotein cholesterol (<40 mg/dL for men and <50 mg/dL in women). Use of antihypertensive, antidiabetic, and lipid-lowering drugs was defined as prescriptions lasting >30 days between 2009 and 2011.

RHF was defined based on previous reports (18, 19). Residuals were calculated using multiple linear regression analysis with eGFR as the dependent variable and age, sex, weight, and height as independent variables. RHF was defined as residuals of eGFR >95th percentile, and a normal renal filtration rate was defined as an eGFR between the 25th and 75th percentiles (20).

Patients were classified based on MetS and renal hyperfiltration status as follows: Non-MetS with NRF (*n* = 2,819,396), Non-MetS with RHF (*n* = 282,992), MetS with NRF (*n* = 730,841), and MetS with RHF (*n* = 71,837). Propensity score matching was performed using age, sex, and the Charlson Comorbidity Index (CCI) to minimize selection bias related to key determinants of adverse clinical outcomes. Matching scores were estimated using a multivariable logistic regression model, and the nearest-neighbor method was used to construct a 1:1:1:1 matched cohort (*n* = 69,638 in each group).

### 2.3 Data collection

Baseline participant demographics collected included age, sex, body mass index (BMI), smoking, alcohol consumption, physical activity, blood pressure, fasting plasma glucose, hemoglobin, serum creatinine, and lipid profiles. Kidney function was assessed via eGFR, calculated using the 2009 Chronic Kidney Disease Epidemiology Collaboration (CKD-EPI) equation (21). Information on individual prescriptions for antidiabetic, antihypertensive, and lipid-lowering medication was collected. Comorbidities were assessed using the ICD-10-based CCI (22). Comorbid conditions were assigned weighted scores based on their impact on overall health, and these scores were then summed to generate the CCI score.

### 2.4 Outcome measures

The primary endpoints of the study were CV events, progression to end-stage kidney disease (ESKD), and all-cause mortality. The CV events included myocardial infarction, ischemic stroke, and death from CV events. Myocardial infarction and ischemic stroke were identified using ICD-10 codes (I21, I22, or I23 for myocardial infarction; I63 or I64 for ischemic stroke) newly issued during hospitalization or after ≥2 outpatient visits. Progression to ESKD was defined as a condition requiring dialysis or kidney transplantation. ESKD was identified using (1) specific renal replacement codes for insurance coverage and/or (2) a procedure code for ≥12 consecutive dialysis sessions, and/or (3) diagnostic or procedure codes for kidney transplantation (13, 23). All-cause death was determined using nationwide mortality information of death certificate data from the National Statistical Office. All patients were followed from January 1, 2011, until death, last health checkup, or December 31, 2018.

### 2.5 Statistical analyses

All continuous data are expressed as the mean ± standard deviation, and categorical data are reported as numbers and percentages within each group. Differences between the groups were compared using analysis of variance and chi-square tests. Outcome incidence rates were calculated by dividing the number of incident cases by the follow-up duration and expressed per 1,000 person-years. Kaplan–Meier plots were used to show the cumulative event rates for CV events, progression to ESKD, and all-cause mortality. Univariable and multivariable Cox regression analysis evaluated the risk of adverse clinical outcomes. Multivariable analysis was adjusted for age, sex, smoking habits, alcohol consumption, physical activity, BMI, CCI, hemoglobin, and low-density lipoprotein cholesterol. As baseline characteristics differed among the four groups in the matched cohort, unmatched variables were included in the multivariate Cox regression model. The interaction term between MetS and RHF was assessed, in addition to the main effects of the fully adjusted models. *p* values <0.05 were used to reject the null hypothesis. All statistical analyses were performed using Statistical Analysis System software (version 9.4; SAS, SAS Institute., Cary, NC, United States).

## 3 Results

### 3.1 Baseline characteristics

Table 1 presents the baseline demographics and clinical characteristics of the matched cohort, categorized by the presence of MetS and RHF. Age, sex, and CCI were well-balanced between groups. The proportions of current smokers, moderate drinkers (≥2 d/week), and individuals with infrequent physical activity (0–2 d/week) were higher in patients with MetS than in those without (*p* < 0.001 for all). The presence of RHF was associated with a higher prevalence of current smoking and frequent drinking in both non-MetS and MetS groups. eGFR levels significantly increased in patients with RHF, whereas MetS status did not affect its levels (eGFR levels of 94 ± 9 vs. 121 ± 18 vs. 92 ± 8 vs. 119 ± 16 mL/min/1.73 m^2^, non-MetS with NRF vs. non-MetS with RHF vs. MetS with NRF vs. MetS with RHF, *p* < 0.001). Patients with MetS and RHF had the highest waist circumference, BMI, systolic blood pressure, fasting blood glucose, and triglyceride levels among the four groups.

**Table 1:** Baseline and clinical characteristics of the propensity score-matched cohort.

Variables	Non-MetS with NRF (*n* = 69,638)	Non-MetS with RHF (*n* = 69,638)	MetS with NRF (*n* = 69,638)	MetS with RHF (*n* = 69,638)	*p*-value
Age, yr	44.8 ± 11.7	44.8 ± 11.7	44.8 ± 11.7	44.8 ± 11.7	1.000
Men, n (%)	50,150 (72.0)	50,152 (72.0)	50,150 (72.0)	50,150 (72.0)	1.000
Waist circumference, cm	79.5 ± 7.9	80.4 ± 8.1	88.6 ± 8.5	90.0 ± 9.1	<0.001
BMI, kg/m^2^	23.3 ± 2.8	23.5 ± 3.0	26.7 ± 3.4	27.1 ± 3.8	<0.001
Smoking					<0.001
Never, n (%)	33,333 (48.0)	32,666 (46.9)	30,517 (43.8)	29,847 (42.9)	
Past, n (%)	12,961 (18.6)	11,871 (17.1)	12,983 (18.6)	11,949 (17.2)	
Current, n (%)	23,286 (33.4)	25,101 (36.0)	26,136 (37.5)	27,841 (40.0)	
Alcohol consumption					<0.001
0–1 d/week, n (%)	47,053 (67.6)	45,317 (65.1)	43,654 (62.7)	41,893 (60.2)	
≥2 d/week, n (%)	22,854 (32.4)	24,321 (34.9)	25,983 (37.3)	27,745 (39.8)	
Physical activity					<0.001
0–2 d, n (%)	32,901 (47.3)	33,032 (47.4)	34,341 (49.3)	34,113 (49.0)	
≥3 d, n (%)	36,736 (52.8)	36,606 (52.6)	35,296 (50.7)	35,525 (51.0)	
Charlson comorbidity index	1.4 ± 1.6	1.4 ± 1.6	1.4 ± 1.6	1.4 ± 1.6	1.000
eGFR, mL/min per 1.73 m^2^	94 ± 9	121 ± 18	92 ± 8	119 ± 16	<0.001
SBP, mm Hg	120 ± 13	121 ± 13	131 ± 13	132 ± 13	<0.001
Hemoglobin, g/dL	14.4 ± 1.5	14.2 ± 1.5	14.7 ± 1.5	14.5 ± 1.5	<0.001
Fasting blood glucose	93 ± 16	93 ± 19	109 ± 30	112 ± 36	<0.001
Total cholesterol, mg/dL	194 ± 34	191 ± 36	205 ± 39	202 ± 42	<0.001
Triglyceride, mg/dL	121 ± 84	121 ± 86	227 ± 148	233 ± 175	<0.001
LDL-cholesterol, mg/dL	115 ± 40	112 ± 39	116 ± 47	113 ± 43	<0.001
HDL-cholesterol, mg/dL	56.2 ± 17.6	56.7 ± 27.9	47.1 ± 17.5	47.2 ± 24.7	<0.001
Lipid-lowering usage, n (%)	66,176 (95.0)	66,053 (94.9)	55,766 (80.1)	54,903 (78.8)	<0.001

Data are expressed as mean ± standard deviation or number (%). MetS, metabolic syndrome; NRF, normal renal filtration; RHF, renal hyperfiltration; n, number; BMI, body mass index; eGFR, estimated glomerular filtration rate; SBP, systolic blood pressure; LDL, low-density lipoprotein; HDL, high-density lipoprotein.

### 3.2 Cumulative event rates of adverse clinical outcomes based on RHF and MetS status

**Figure 2:**
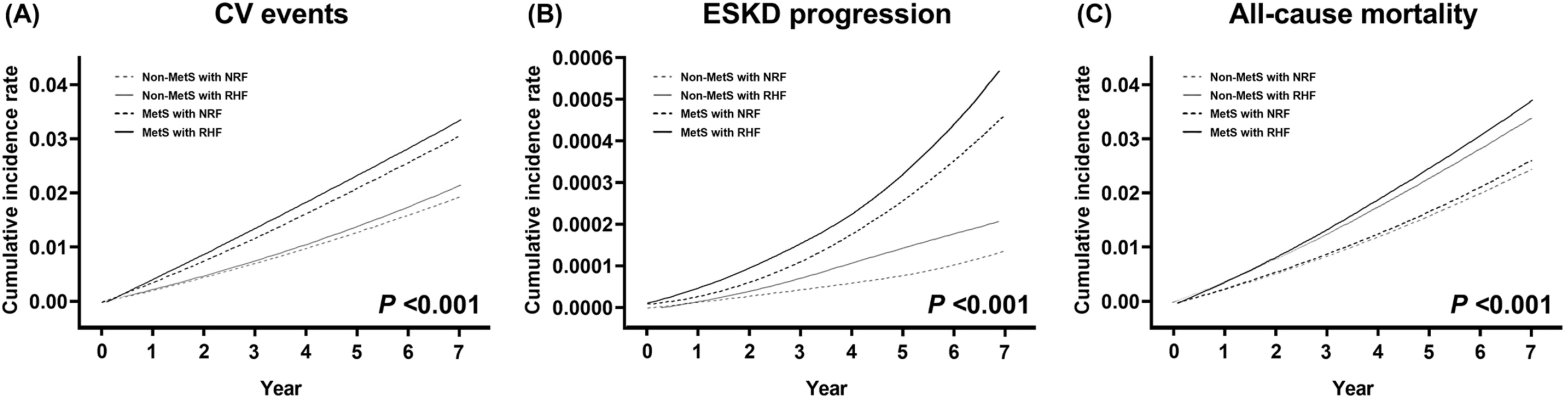
Cumulative event rates for adverse clinical outcomes according to metabolic syndrome and renal hyperfiltration status. **(A)** Cardiovascular events, **(B)** progression to end-stage kidney disease, and **(C)** all-cause mortality. *p* values are calculated by log-rank test. MetS, metabolic syndrome; NRF, normal renal filtration; RHF, renal hyperfiltration.

During a mean follow-up of 6.9 years, CV events, progression to ESKD, and all-cause mortality occurred in 6,659, 104, and 5,165 patients, with incidence rates of 3.48, 0.05, and 2.67 per 1,000 person-years, respectively. Cumulative incidence rates of these outcomes significantly differed across the four groups (*p* < 0.001) (Figure 2). The non-MetS with NRF group had the lowest, and the MetS with RHF group had the highest incidence rates for all three outcomes.

### 3.3 Adverse clinical outcome risks based on RHF and MetS status

Table 2 shows the number of events, incidence rates, and observed hazard ratios (HRs) for predefined outcomes based on RHF and MetS presence. Univariable Cox analysis showed that the presence of MetS or RHF was associated with increased risks of adverse clinical outcomes. The highest risks were observed in patients with both MetS and RHF [unadjusted HR = 1.76, 95% confidence interval (CI) = 1.65–1.89; unadjusted HR = 3.90, 95% CI = 1.95–7.81; and unadjusted HR = 1.52, 95% CI = 1.41–1.65 for CV events, progression to ESKD, and all-cause mortality, respectively]. In the multivariable Cox analysis, non-MetS patients with RHF did not show an increased risk of CV events compared to non-MetS patients with NRF. However, the presence of MetS alone was associated with an increased CV event risk (adjusted HR = 1.31, 95% CI = 1.23–1.39), and this risk was further amplified when combined with RHF, with a significant interaction observed (adjusted HR = 1.44, 95% CI = 1.35–1.55, P for interaction = 0.047). Similarly, RHF alone did not increase the risk of ESKD progression in non-MetS patients; however, the risk was significantly increased in patients with MetS and NRF (adjusted HR = 3.02, 95% CI = 1.56–5.87) and was greatest in those with both MetS and RHF (adjusted HR = 3.23, 95% CI = 1.61–6.47). All-cause mortality risk significantly increased in non-MetS patients with RHF (adjusted HR = 1.26, 95% CI = 1.18–1.35) and was highest in patients with MetS and RHF (adjusted HR = 1.41, 95% CI = 1.31–1.52). No significant interaction between MetS and RHF was observed for ESKD progression or all-cause mortality (P for interaction = 0.457 and 0.584).

**Table 2:** Hazard ratios of clinical outcomes based on the presence of RHF and MetS in the matched cohort.

Variables	No. of events	Incidence rates	Unadjusted HR (95% CI)	*P*-value	Adjusted HR^a^ (95% CI)	*P*-value	*P* for interaction
CV events							
Non-MetS with NRF	1,226	2.55	Reference		Reference		0.047
Non-MetS with RHF	1,359	2.84	1.11 (1.03–1.20)	0.007	1.01 (0.94–1.09)	0.847	
MetS with NRF	1,933	4.04	1.59 (1.48–1.70)	<0.001	1.31 (1.23–1.39)	<0.001	
MetS with RHF	2,141	4.50	1.76 (1.65–1.89)	<0.001	1.44 (1.35–1.55)	<0.001	
Progression to ESKD							
Non-MetS with NRF	13	0.03	Reference		Reference		0.457
Non-MetS with RHF	17	0.04	1.40 (0.62–3.15)	0.416	1.46 (0.73–2.92)	0.288	
MetS with NRF	32	0.07	3.10 (1.52–6.32)	0.002	3.02 (1.56–5.87)	0.001	
MetS with RHF	42	0.09	3.90 (1.95–7.81)	<0.001	3.23 (1.61–6.47)	0.001	
All-cause mortality							
Non-MetS with NRF	1,036	2.14	Reference		Reference		0.584
Non-MetS with RHF	1,433	2.97	1.39 (1.28–1.50)	<0.001	1.26 (1.18–1.35)	<0.001	
MetS with NRF	1,123	2.32	1.08 (1.00–1.18)	0.060	1.15 (1.07–1.23)	<0.001	
MetS with RHF	1,573	3.26	1.52 (1.41–1.65)	<0.001	1.41 (1.31–1.52)	<0.001	

Incidence rates are expressed as incidence per 1,000-person-years. ^a^Adjusted for the following variables: age, body mass index, sex, Charlson comorbidity index, smoking, alcohol consumption, physical activity, hemoglobin, and low-density lipoprotein-cholesterol. No, number; HR, hazard ratio; CI, confidence interval; CV, cardiovascular; MetS, metabolic syndrome; NRF, normal renal filtration; RHF, renal hyperfiltration; ESKD, end-stage kidney disease.

The impact of MetS and RHF on clinical outcomes was examined in the entire study population. Baseline demographics and clinical characteristics of the entire cohort are presented in Supplementary Table 1. Multivariable Cox analysis revealed results consistent with those in the propensity score-matched cohort. The presence of RHF alone was not associated with an increased risk of CV events or ESKD progression, and the risk of all-cause mortality significantly increased (Supplementary Table 2). MetS with RHF synergistically increased CV risk (adjusted HR = 1.45, 95% CI = 1.38–1.51, P for interaction = 0.002). Risks of ESKD progression (adjusted HR = 2.22, 95% CI = 1.58–3.11) and all-cause mortality (adjusted HR = 1.45, 95% CI = 1.38–1.53) were highest in patients with MetS and RHF compared to non-MetS patients with NRF.

### 3.4 Adverse clinical outcome risks based on the number of MetS components and RHF

**Figure 3:**
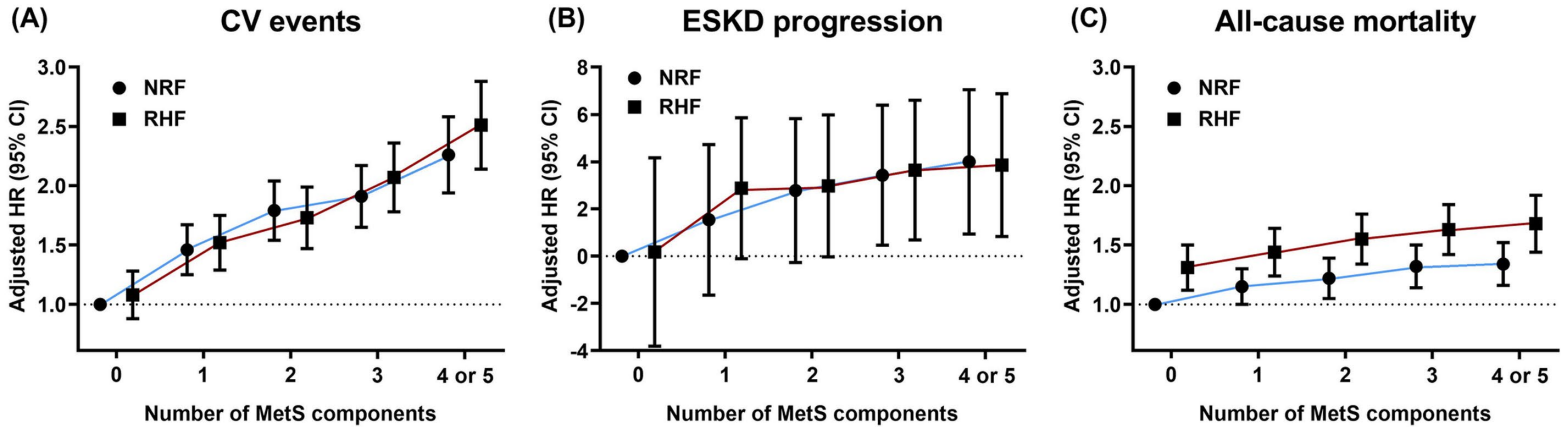
Hazard ratios for adverse clinical outcomes based on the number of metabolic risk factors and renal hyperfiltration presence. Dots and bars indicate adjusted hazard ratios and 95% confidence intervals, respectively. **(A)** cardiovascular events, **(B)** progression to ESKD, and **(C)** all-cause mortality. The HRs for ESKD progression were calculated using log2-transformed values. NRF, normal renal filtration; RHF, renal hyperfiltration; HR, hazard ratio; CV, cardiovascular; ESKD, end-stage kidney disease.

Figure 3 illustrates observed HRs for the predefined outcomes based on the number of MetS components and the presence of RHF in the matched cohort. The CV event risk increased with the number of dysfunctional MetS components regardless of RHF status. Patients with RHF exhibited a more rapid increase in HRs for CV events than those with NRF when more than two dysfunctional metabolic components were present. Accordingly, a significant interaction was observed between the number of MetS components and RHF presence for CV events (P for interaction = 0.019). A consistent trend of increasing adjusted HRs for ESKD progression and all-cause mortality was observed in patients with NRF and RHF, with no significant interactions between RHF and MetS presence (P for interaction = 0.900 and 0.616, respectively). Patients with RHF with more than three dysfunctional MetS components showed significantly increased ESKD progression and mortality risks, even in the absence of dysfunctional MetS components.

## 4 Discussion

Using a nationwide population-based cohort from the NHIS database, our study demonstrated that RHF increased the risks of CV events, progression to ESKD, and all-cause mortality when combined with MetS. The coexistence of RHF and MetS was associated with a synergistic increase in CV event risk, whereas this association was not observed in the absence of MetS, indicating that the association between RHF and CV event risk depends on MetS status. RHF was also linked to higher risk of all-cause mortality, even in the absence of MetS. However, among individuals without MetS, RHF was not significantly associated with CV events or ESKD progression risks. Overall, these findings suggest that the clinical significance of RHF varies according to MetS status, with RHF alone having varying implications depending on the clinical outcome.

Recent studies indicate that MetS and cardiovascular and kidney disease should not be regarded as isolated disease entities but rather as interrelated components of a broader clinical spectrum (24). Among the various pathophysiological factors, inflammation represents a central mechanistic pathway linking MetS, RHF, and obesity. Dysfunctional adipose tissue, particularly visceral fat, is a major source of proinflammatory mediators that drive systemic insulin resistance, oxidative stress, and lipid disturbance (25, 26). These processes can induce renal structural damage and glomerular hyperfiltration, which subsequently accelerate glomerulosclerosis, tubular inflammation, and fibrosis, thereby connecting metabolic stress with adverse renal outcomes. In addition, systemic inflammation exacerbates endothelial dysfunction and activates the sympathetic nervous system, both of which contribute to the development of adverse cardiovascular outcomes (27). Therefore, chronic low-grade inflammation driven by obesity-related adipose dysfunction may explain the synergistic association of MetS and RHF with long-term adverse clinical outcomes.

All MetS components are significant risk factors for CV diseases (28). This study similarly showed that an increasing number of dysfunctional MetS components increased CV event risk regardless of RHF status. Patients with RHF exhibited a steeper increase in CV event risk per additional MetS component than those with NRF, with a statistically significant interaction. These findings indicate that RHF may act as a sensitizer, amplifying the detrimental effects of metabolic derangements on CV health. This synergistic interaction may be explained by shared pathophysiological mechanisms between MetS and RHF, such as renin-angiotensin system activation and endothelial dysfunction (10, 29). We hypothesize that these mechanisms collectively accelerate vascular damage and atherosclerosis, ultimately contributing to worse CV outcomes.

Previous studies have shown that RHF is independently associated with rapid kidney function decline (30, 31). However, in this study, non-MetS patients with RHF did not have an increased risk of ESKD progression, which was elevated only when RHF coexisted with MetS. Furthermore, among patients with RHF, ESKD progression risk increased in the presence of three or more dysfunctional MetS components. These findings imply that RHF alone is not strongly linked to ESKD progression risk; however, the risk becomes clinically significant when MetS is present. Therefore, we suggest that the clinical significance of RHF depends on MetS status and highlights the need to prioritize risk stratification based on their coexistence.

Several studies have demonstrated that patients with RHF are at a higher risk of all-cause mortality; however, none have examined the clinical implications of RHF in relation to MetS status (19, 32). In this study, patients with MetS and RHF had the most pronounced increase in mortality risk, with a clear gradient depending on metabolic dysfunction severity. These findings suggest the cumulative effect of RHF and MetS on mortality risk. RHF was associated with significantly elevated all-cause mortality risk—even in the absence of MetS—including in individuals with few dysfunctional MetS components. This suggests that RHF alone contributes to increased all-cause mortality risk, and the potential for death from MetS-unrelated complications, such as infections, may be substantially higher in non-MetS patients with RHF.

Effective strategies exist to target glomerular hypertension and suppress the renin-angiotensin system. Angiotensin-converting-enzyme inhibitors or angiotensin-receptor blockers decrease glomerular pressure and promote RHF restoration (33). Therefore, this study supports the idea that renin-angiotensin inhibitors can be beneficial in reducing the risk of CV events, ESKD progression, and all-cause mortality in patients with RHF. However, the combination of MetS and RHF was associated with the highest risk of adverse clinical outcomes across all patient categories. These findings suggest that targeted treatment against RHF alone is insufficient and that simultaneous improvement of MetS is necessary for optimal patient care. In this perspective, sodium-glucose cotransporter-2 inhibitors can be more advantageous for patients with MetS and RHF, given their high effectiveness in improving MetS status, reducing body weight, and decreasing glomerular filtration (24, 34, 35). In addition, glucagon-like peptide-1 receptor agonists (GLP-1RAs) not only facilitate weight reduction but also confer renoprotective (36, 37), cardioprotective (38, 39), and anti-inflammatory effects (40). These multifaceted benefits may be especially relevant for patients with coexisting MetS and RHF, simultaneously targeting both metabolic and kidney-cardiovascular risk factors.

This study had certain limitations. First, selection bias was possible, as healthier individuals are more prone to receive health checkups, whereas those with serious comorbidities may not. Second, although anthropometric measurements were adjusted to define RHF, muscle mass could still influence filtration status, and data on muscle mass or measured GFR were unavailable in the Korean national health screening dataset. Third, information on albuminuria was not collected in this study. Finally, although baseline eGFR levels differed based on RHF and MetS status, assessing the rate of renal function decline would have been more informative. However, regular follow-up data on serum creatinine levels were lacking in this cohort.

In conclusion, our findings demonstrate that the clinical significance of RHF varies according to MetS status. RHF was significantly associated with an increased risk of CV events and ESKD progression in the presence of MetS. The risks of CV events, ESKD progression, and all-cause mortality were highest in patients with both MetS and RHF. Their coexistence resulted in a synergistic increase in CV risk. These findings offer new insights into risk stratification for patients with MetS and RHF and may inform strategies to reduce adverse clinical outcomes.

## Data Availability

The datasets generated and/or analyzed during the current study are available from the NHIS database upon request. Requests to access these datasets should be directed to hwanghsne@gmail.com.
