# Supplementary material for: Renal hyperfiltration with and without metabolic syndrome: differential implications for cardiovascular events, kidney failure, and mortality
Source: Front Nutr. 2025 Oct 14;12:1652372. doi: 10.3389/fnut.2025.1652372 (PMC12558771; doi:10.3389/fnut.2025.1652372)
Supplement: Supplementary file 1 [file Table_1.pdf]

Supplementary Table 1. Baseline characteristics and clinical characteristics of the whole study population

	Non-MetS with NRF (n=2,817,396)	Non-MetS with RHF (n=282,992)	MetS with NRF (n=730,841)	MetS with RHF (n=71,837)	*P* value
Age, year	45.7±12.2	39.4±11.3	52.2±12.2	44.8±11.7	< 0.001
Men, n (%)	1,581,044 (56.1)	158,972 (56.2)	449,165 (61.5)	51,797 (72.1)	< 0.001
BMI, kg/m^2	78.0±8.3	78.4±9.0	87.5±8.3	90.0±9.1	< 0.001
Waist circumference, cm	23.0±2.9	23.1±3.2	26.1±3.2	27.1±3.8	< 0.001
Smoking					
Never, n (%)	1,657,742 (58.8)	162,498 (57.4)	387,162 (53.0)	30,718 (42.8)	< 0.001
Past, n (%)	428,314 (15.2)	36,226 (12.8)	133,844 (18.3)	12,313 (17.1)	
Current, n (%)	731,220 (26.0)	84,265 (29.8)	209,805 (28.7)	28,805 (40.1)	
Alcohol consumption					
0-1 day/week, n (%)	2,045,275 (72.6)	198,235 (70.1)	493,765 (67.6)	43,137 (60.1)	< 0.001
≥2 days/week, n (%)	771,968 (27.4)	84,754 (30.0)	237,031 (32.4)	28,700 (40.0)	
Physical activity					
0-2 days, n (%)	1,335,435 (47.4)	135,246 (47.8)	352,112 (48.2)	35,207 (49.0)	< 0.001
≥3 days, n (%)	1,481,910 (52.6)	147,746 (52.2)	378,711 (51.8)	36,630 (51.0)	
Charlson comorbidity index	1.1±1.3	1.0±1.2	1.8±1.8	1.6±1.8	< 0.001
eGFR, ml/min per 1.73 m^2	94±9	124±17	89±9	119±16	< 0.001
SBP, mm Hg	119±13	119±13	131±14	132±14	< 0.001
Hemoglobin, g/dl	14.0±1.6	13.9±1.7	14.4±1.6	14.6±1.6	< 0.001

Fasting blood glucose	93±16	91±17	111±30	113±37	< 0.001
Total cholesterol, mg/dl	193±34	187±35	205±40	203±42	< 0.001
Triglyceride, mg/dl	113±77	112±83	213±141	234±178	< 0.001
LDL-cholesterol, mg/dl	114±40	108±38	117±46	114±43	< 0.001
HDL-cholesterol, mg/dl	57±17	58±29	48±16	47±25	< 0.001
Lipid-lowering usage, n (%)	2,668,073 (94.7)	273,749 (96.7)	529,851 (72.5)	56,405 (78.5)	< 0.001

Data are presented as mean ± standard deviation or number (%).

MetS, metabolic syndrome; NRF, normal renal filtration; RHF, renal hyperfiltration; n, number; BMI, body mass index; eGFR, estimated glomerular filtration rate; SBP, systolic blood pressure; LDL, low-density lipoprotein; HDL, high-density lipoprotein.

Supplementary Table 2. Hazard ratios of clinical outcomes based on the presence of RHF and MetS in the whole study population

	No. of events	Incidence rates	Unadjusted HR (95% CI)	*P* value	Adjusted HR[a] (95% CI)	*P* value	*P* for interaction
CV events							
Non-MetS with NRF	54,890	2.83	Reference		Reference		0.002
Non-MetS with RHF	3,362	1.72	0.61 (0.59 – 0.63)	<0.001	1.02 (0.98 – 1.05)	0.377	
MetS with NRF	33,707	6.81	2.41 (2.38 – 2.45)	<0.001	1.30 (1.28 – 1.32)	<0.001	
MetS with RHF	2,213	4.51	1.60 (1.53 – 1.67)	<0.001	1.45 (1.38 – 1.51)	<0.001	
Progression to ESKD							
Non-Mets with NRF	820	0.04	Reference		Reference		0.270
Non-Mets with RHF	69	0.04	0.78 (0.60 – 1.03)	0.075	0.97 (0.72 – 1.31)	0.851	
MetS with NRF	641	0.13	3.26 (2.92 – 3.63)	<0.001	1.77 (1.52 – 2.07)	<0.001	
MetS with RHF	59	0.12	3.08 (2.34 – 4.04)	<0.001	2.22 (1.58 – 3.11)	<0.001	
All-cause mortality							
Non-MetS with NRF	47,576	2.43	Reference		Reference		0.351
Non-MetS with RHF	3,814	1.94	0.80 (0.77 – 0.82)	<0.001	1.26 (1.22 – 1.31)	<0.001	
MetS with NRF	21,022	4.16	1.71 (1.69 – 1.74)	<0.001	1.12 (1.10 – 1.14)	<0.001	
MetS with RHF	1,653	3.32	1.37 (1.30 – 1.44)	<0.001	1.45 (1.38 – 1.53)	<0.001	

Incidence rates are expressed as incidence per 1000-person years.

[a] Adjusted for the following variables: age, body mass index, sex, Charlson comorbidity index, smoking, alcohol consumption, physical activity, hemoglobin, and low-density lipoprotein cholesterol.

No, number; HR, hazard ratio; CI, confidence interval; CV, cardiovascular; MetS, metabolic syndrome; NRF, normal renal filtration; RHF, renal hyperfiltration; ESKD, end-stage kidney disease.
